# Associations between sociodemographic factors, experience of death and dying, and death literacy: A nationally representative cross-sectional study

**DOI:** 10.1177/26323524261473937

**Published:** 2026-09-03

**Authors:** Melanie F. J. Diggle, Therese Johansson, Joanna M. Davies, Kate Heaps, Katherine E. Sleeman

**Affiliations:** 1 Cicely Saunders Institute of Palliative Care, Policy & Rehabilitation, Florence Nightingale Faculty of Nursing, Midwifery & Palliative Care, 4616 King’s College London, London, UK; 2 South East London Integrated Care System, London, UK

**Keywords:** death literacy, end-of-life care, palliative care, bereavement, public health

## Abstract

**Background:**

Death literacy is the knowledge and skills that enable people to navigate and act on end-of-life issues, and underpins public health approaches to palliative and end-of-life care. Nationally representative evidence on factors associated with death literacy remains limited.

**Objectives:**

To measure death literacy in adults and examine associations with sociodemographic characteristics and experiential exposure.

**Design and methods:**

A cross-sectional online survey of a nationally representative United Kingdom (UK) sample (n = 2,106), completing the Death Literacy Index-Revised (DLI-R) and providing sociodemographic and contextual information. Weighted data were analysed using descriptive statistics and linear regression.

**Results:**

The mean DLI-R score was 6.01 (SD = 1.85). The Experiential Knowledge scale had the highest score (7.15, SD = 1.98), while Accessing Help had the lowest (5.16, SD = 2.64). Compared to no experiential exposure, personal/family exposure (B = 0.69, 95% CI 0.53, 0.85) and professional/volunteer exposure (B = 1.38, 95% CI 1.14, 1.63) were significantly associated with higher DLI-R scores. Adults aged 25–34 scored higher than those aged 65+ (B = 0.45, 95% CI 0.18, 0.71). Compared to the married group, the ‘single and never married’ group (B = -0.61, 95% CI -0.82, -0.41) had lower DLI-R scores. There were ethnic differences in scores, with Black/Black British people found to have higher death literacy compared to the reference group of White/White British people (B = 0.71, 95% CI 0.35, 1.08).

**Conclusion:**

This study identified gaps in UK death literacy with findings highlighting that death literacy is shaped mostly by personal and professional experience. Public health approaches that promote community learning, and clearer support pathways, may help address these gaps. Further work is needed to identify effective ways to strengthen death literacy at a population level.

## Plain language summary

Death literacy involves understanding end-of-life options, knowing how to access help, and feeling confident supporting others who are dying or grieving. This study explored the death literacy among people in the United Kingdom (UK). 2,106 adults across the UK took part in an online survey. The survey asked about peoples’ knowledge and experiences of dying, death and bereavement using a scale called Death Literacy Index-Revised (DLI-R), as well as background information such as their age and ethnicity. Overall, people in the UK scored around 6 out of 10 on the DLI-R scale. The highest scores related to knowledge and skills gained through personal experiences, while the lowest scores were in knowing how to find and access help in the community. People who had personally cared for someone at the end of life, or who had worked or volunteered in a setting involving death or bereavement, had higher scores than those with no such experience. Marital status also mattered, with people who were single and who had never married scoring lower than married people. Adults aged 25–34 reported slightly higher death literacy scores than older adults. Ethnicity also played a role, with Black and Black British people scoring higher compared to other groups such as White and White British people. Death literacy scores were not influenced by gender or education level. This study shows that personal and professional experiences with death and dying play an important role in shaping people’s knowledge, skills, and confidence around end-of-life care. Approaches that encourage people to learn from others’ experiences, support community involvement, and make it easier to find information and access services may help people better support one another through dying, death, and bereavement.

## Background

Death literacy refers to the knowledge, skills, and social capacities that enable individuals and communities to understand and access care and support options related to dying, death, and bereavement within their cultural and social contexts.^1,2^ It is increasingly recognised as a central component of public health approaches to palliative and end-of-life care, reflecting the understanding that dying and bereavement are not solely medical issues but matters of collective concern that require community capability and shared responsibility.^3^ Conceptually, death literacy is grounded in experiential competence; people develop the ability to engage with death through encounters with serious illness and participation in community practices surrounding dying and grief.^1,4,5^

The construct of death literacy was first articulated through community-based research in Australia exploring the experiences of families supporting individuals to die at home.^4,5^ These studies demonstrated how practical skills, local knowledge, and informal networks enabled communities to participate meaningfully in end-of-life care. In this sense, death literacy is analogous to health literacy; both involve navigating complex systems, exercising informed choice, and engaging in dialogue with professionals and peers.^1,6^ The key dimensions of death literacy encompass factual and procedural knowledge (e.g., managing administrative tasks after a death), interpersonal communication (e.g., discussing dying with others), and collective or community skills (e.g., drawing on support groups).^7^ Higher levels of death literacy are associated with earlier engagement with end-of-life services, greater caregiving confidence, and stronger community capacity to support dying and bereavement.^8^

To operationalise the construct, Leonard et al.^8^ developed the Death Literacy Index (DLI) through a mixed-methods approach which integrated existing theoretical conceptualisations of death literacy^1^ with relevant empirical measures, alongside input from end-of-life care professionals. The DLI is currently the only validated instrument designed to measure death literacy as a multidimensional construct. Validation work by the original developers supported the DLI’s reliability and factor structure, supporting its use in both population-based and intervention studies.^2,8^ In the United Kingdom (UK), the DLI has demonstrated strong psychometric properties, with high internal consistency and good convergent and discriminant validity; this work has also provided an initial national DLI score benchmark.^9^

Since its development, the DLI has been adapted and validated across various cultural contexts, including countries in Europe and Asia.^10–13^ However, cultural adaptation work revealed challenges in aligning item wording with the cultural and systemic contexts surrounding death. In response, the original English-language DLI was refined in 2024 to enhance readability and accessibility, creating the Death Literacy Index–Revised (DLI-R), which has been validated in Australia with a preserved underlying factor structure compared to the original instrument.^14^

Despite international research progress, there remains a lack of nationally representative data examining how death literacy varies across sociodemographic groups and end of life experiential exposures in the UK. Addressing this gap using the DLI-R is critical, as sociodemographic factors, such as age, alongside end-of-life experiences, have been shown to be associated with death literacy.^8,9^ Understanding variation across population groups can therefore help to identify opportunities to strengthen death literacy and inform public health strategies aimed at improving community capacity for end-of-life care.^3,15^

Therefore, the aim of this study was to measure death literacy in the UK and explore the sociodemographic and end-of-life experiential exposure variables associated with death literacy.

## Methods

### Study design

This cross-sectional study used data on death literacy, sociodemographic characteristics, and exposure to end-of-life experiences from a nationally representative survey of UK adults administered by Focaldata, a UK-based polling company that administers and distributes surveys to the general public. The study reporting adheres to the Checklist for Reporting Results of Internet E-Surveys (CHERRIES)^16^ (Supplementary Appendix 1).

### Population and sampling

Respondents were sampled by Focaldata, which uses a network of third-party online panel providers, i.e., companies and organisations that recruit and maintain pools of people who can be invited to complete surveys for research purposes. These panels enable companies such as Focaldata to achieve population representativeness across key sociodemographic characteristics through use of a stratified sample with post-stratification weighting. In this case, the strata were based on age, gender, region, educational attainment, marital status and ethnicity. Inclusion criteria were being aged ≥18 years and resident in the UK. No exclusion criteria were used. There was no target sample size set by the research team; rather, Focaldata recruited participants until representativeness was achieved (commonly around 2,000 participants).

### Procedure

#### Survey design

The survey included the DLI-R (presented in original wording and order, see Supplementary appendix 2) and additional questions on sociodemographic characteristics and end-of-life experiences. The DLI-R items and survey questions were presented across 21 pages in total. Adaptive questioning was not used. Forced responses were employed to prevent missing data, and respondents could navigate backwards to review and amend their answers.

#### Recruitment and data collection

The survey was administered via Focaldata and advertised through a network of third-party panel providers. Eligible panel members were invited to participate via email and accessed the closed survey through a unique, single-use link. Before commencing, respondents were informed about the estimated survey length, purpose (including the focus on end-of-life care experiences), voluntary nature of participation, data handling procedures, and incentives. Informed consent was provided electronically by proceeding with the survey. The survey could be completed on a desktop, tablet, or mobile device. Participation was voluntary; respondents retained the option to withdraw at any time by closing their browser. Data were collected between 21–25 March 2025. Panel-specific reimbursements (e.g. monetary payment or vouchers) were managed by panel providers upon survey completion.

#### Quality assurance and data management

Automated checks were implemented by Focaldata to identify and exclude low-quality responses indicative of disengagement or fraudulent behaviour. Survey completion times were monitored to detect implausibly rapid responses, i.e., less than one-third of the median completion time. Screening was conducted for straight-lining, i.e., providing the same response option across multiple items irrespective of content. Responses failing these checks were removed. Duplicate responses were prevented through panel-based system user registration, retention of cookies when accessing the survey, and IP address checks. Log-file analysis, a method for examining system-generated records of how users interact with a survey, was not undertaken. Focaldata conducted initial data processing and quality checks before sharing the fully anonymised dataset with the research team; the research team had no access to identifiable participant information at any stage.

### Measures

#### Death literacy

Death literacy was measured using the DLI-R,^14^ a 29-item instrument comprising four main scales: Practical Knowledge, Experiential Knowledge, Factual Knowledge, and Community Knowledge (see Supplementary Appendix 2). The Practical Knowledge scale contains two sub-scales which assess people’s confidence in providing practical, hands-on care and physical support to someone at the end of life respectively. The Experiential Knowledge scale reflects knowledge and skills developed through personal encounters with death, bereavement, or serious illness. The Factual Knowledge scale examines familiarity with factual and procedural matters following a death. The Community Knowledge scale comprises two sub-scales, the first capturing awareness of how to navigate formal services, and the second, community-based resources related to end-of-life care and bereavement. The DLI-R is a revised version of the DLI, refined for improved readability. Unlike the original DLI,^9^ the DLI-R has not yet been formally validated with a UK population. However, it has high content equivalence with the original instrument, and the underlying constructs, scales, and item meanings remain unchanged.^14^

DLI-R items are rated using a 5-point Likert scale (ranging 1-5). In adherence to the standard DLI scoring approach,^2,14^ scores are calculated for each scale and sub-scale using transformed mean scores. Raw item responses are summed, adjusted for the minimum possible value, divided by the scale range, and rescaled (range 0–10) to enable comparability across scales with a variable number of items. A total DLI-R score is then calculated as a composite score, with higher DLI-R scores indicating greater overall death literacy.^8^

#### Sociodemographic variables

Sociodemographic data were collected on age (using Focaldata default groupings: 18–24, 25–34, 35–44, 45–54, 55–64, 65+ years), gender (male, female), ethnicity (White/White British, Asian/Asian British, Black/Black British, Mixed, Other), marital status (married, living with a partner, in a relationship but not living together, single/never married, divorced/separated, widowed, prefer not to say), and educational attainment (below degree level, degree level or above).

#### Experiential exposure variable

Exposure to end-of-life experiences was measured using a multi-select question informed by previous online surveys exploring death literacy.^10,13^ Responses were recoded into three mutually exclusive categories.

1. Personal/Family Exposure: Respondents who selected “I have a life-limiting or terminal illness”, “Someone close to me has a life-limiting or terminal illness”, and/or “I or someone close to me has used palliative care or hospice services”.
2. Professional/Volunteer Exposure: Respondents who selected “I am a professional working with people who may be in the last few years of life” and/or “I am a volunteer working with people who may be in the last few years of life”.
3. No Exposure: Respondents who selected none of the responses above

Where respondents reported both personal/family and professional/volunteer exposure, they were assigned to the professional/volunteer category. This does not imply greater importance of professional exposure over personal exposure, rather, it was done to maintain mutually exclusive categories for the analysis.

### Analysis

Analyses were conducted in IBM SPSS Statistics (version 29).^17^ Descriptive statistics are presented for the weighted and unweighted data, summarising independent variables using counts and percentages and DLI-R scores using means and standard deviations.

Using the weighted data, multiple linear regression models were used to examine associations between independent variables and DLI-R scores. Three sets of models were specified: (1) unadjusted models for each independent variable; (2) minimally adjusted models, adjusting for age and gender; and (3) a fully adjusted model adjusting for all independent variables simultaneously.^18^ Robust standard errors were applied to account for potential heteroskedasticity in the weighted survey data.^19^ Reference categories were selected based on the largest group sizes to maximise statistical stability and interpretability of model estimates. Results are presented as unstandardised coefficients (B) with 95% confidence intervals (CI) and statistical significance set at p≤0.05.

To assess the robustness of associations and ensure that findings were not driven by a small subset of respondents with exceptionally high death literacy, in a sensitivity analysis, we reran the main analysis excluding participants who obtained the maximum DLI-R score.

### Ethics

The survey was administered by Focaldata, a UK-based polling company. Participants were recruited from online panels designed for this purpose and consented to be contacted about taking part in survey research. All participants provided informed consent to take part in the survey by clicking ‘proceed’ after being informed of survey details, including questions about end-of-life care experiences. Because the research team only had access to fully anonymised data provided by Focaldata and had no contact with participants, ethical approval was not required, in accordance with King’s College London’s ethics policy. This is in keeping with previous research using data from online polling companies.^20,21,22^

## Results

### Descriptive statistics

In total, 4,188 respondents indicated interest in the survey. Of these, 1,847 (44%) were screened out by Focaldata due to the quota for their demographic sample having been filled. Overall, 2,341 respondents consented to participate and started the survey, Of these, 133 (5.6%) did not complete the survey and 102 (4.4%) were removed due to concerns regarding quality, with 2,106 complete responses included for analysis. It was this sample of 2,106 anonymised responses that Focaldata shared with the research team. Table 1 presents the sample characteristics and unweighted and weighted means and standard deviations for DLI-R scores across all sociodemographic and end of life experience exposure variables.

**Table 1.** Sample characteristics and DLI-R scores across groups (n=2,106).

​	Unweighted				Weighted			
	n	%	DLI-R score	SD	n	%	DLI-R score	SD
Age Group								
18-24	169	8.0	5.71	1.61	232	11.0	5.68	1.58
25-34	379	18.0	6.59	1.83	358	17.0	6.51	1.85
35-44	352	16.7	6.01	2.05	337	16.0	5.98	2.05
45-54	377	17.9	6.00	1.97	358	17.0	5.97	1.94
55-64	366	17.4	5.95	1.81	337	16.0	5.92	1.75
65+	463	22.0	5.93	1.74	484	23.0	5.92	1.74
**Gender**								
Male	1009	47.9	6.03	1.86	1032	49.0	5.98	1.84
Female	1097	52.1	6.09	1.88	1074	51.0	6.04	1.85
**Ethnicity**								
White or White British	1822	86.5	6.00	1.86	1811	86.0	5.99	1.85
Mixed/Multiple Background	35	1.7	5.65	1.79	42	2.0	5.67	1.73
Black or Black British	112	5.3	7.16	1.66	63	3.0	7.24	1.64
Asian or Asian British	122	5.8	6.11	1.86	147	7.0	6.04	1.83
Other	15	0.7	5.50	1.79	42	2.0	5.50	1.55
**Educational Attainment**								
Below degree qualifications	1334	63.3	5.89	1.85	1411	67.0	5.87	1.82
Degree level or above qualifications	772	36.7	6.35	1.87	695	33.0	6.29	1.86
**Marital Status**								
Single and never married	465	22.1	5.64	1.87	498	23.6	5.63	1.83
In a relationship, but not living together	91	4.3	5.45	1.70	98	4.6	5.45	1.68
Living with a partner but not married	270	12.8	5.90	1.74	274	13.0	5.88	1.71
Married	1029	48.9	6.36	1.89	979	46.5	6.31	1.89
Divorced or separated	161	7.6	5.83	1.61	157	7.4	5.86	1.59
Widowed	83	3.9	6.45	1.85	90	4.3	6.28	1.85
Prefer not to say	7	0.3	4.47	1.59	11	0.5	4.99	1.79
**Exposure to End-of-Life Experiences**								
Personal/Family Exposure	568	27.0	6.47	1.64	26.8	564	6.40	1.64
Professional/Volunteer Exposure	223^*^	10.6	7.24	1.66	10.5	221^*^	7.16	1.66
None of these	1315	62.4	5.68	1.88	62.7	1321	5.66	1.85

^*^Of these, 92 respondents had both personal/family exposure and professional/volunteer exposure.

**Figure 1.**
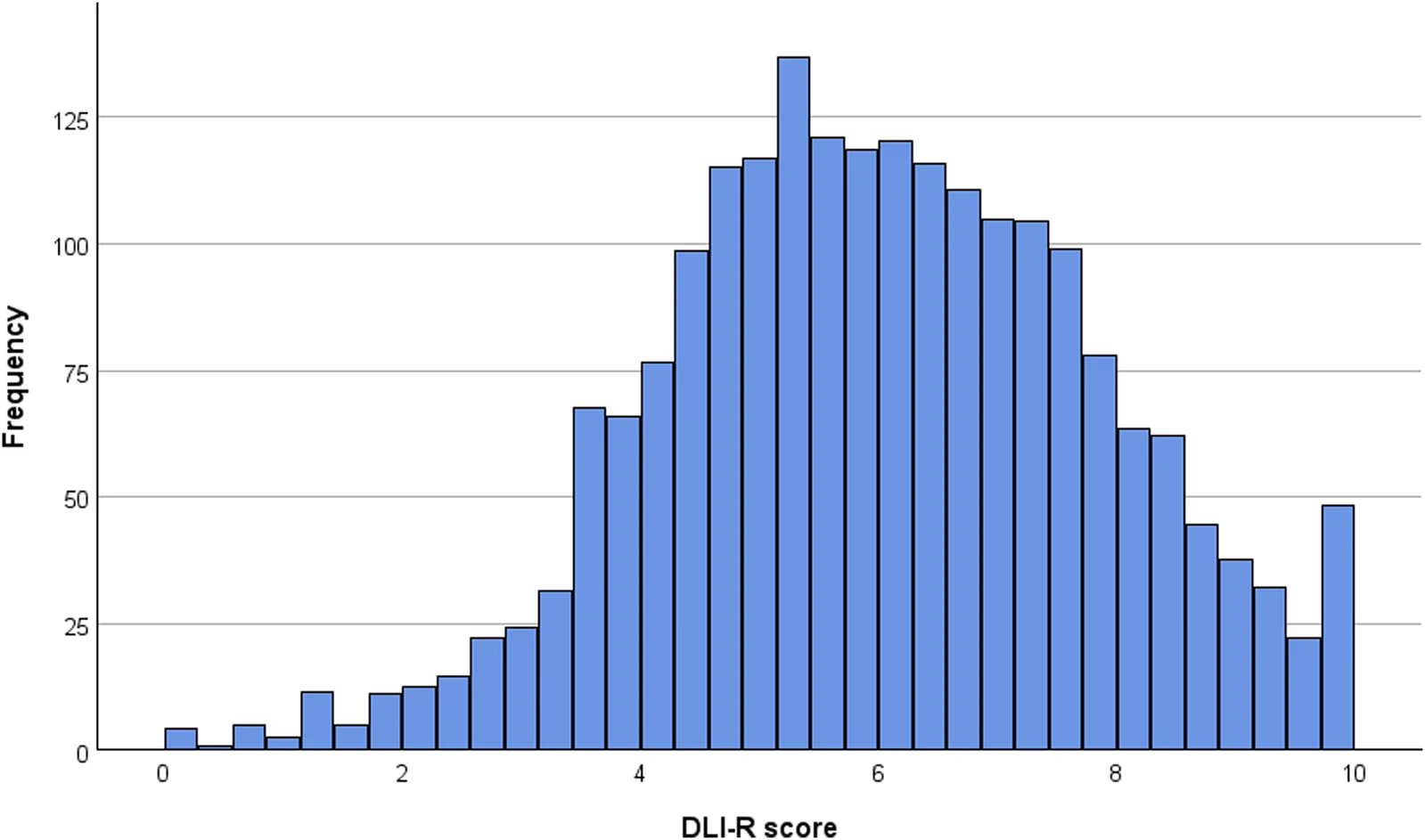
Distribution of DLI-R scores.

The mean total DLI-R score in the weighted sample was 6.01 (SD = 1.85), indicating moderate overall levels of death literacy. As shown in Figure 1, total DLI-R scores were normally distributed for the weighted sample. As presented in Table 2, mean scores varied across the DLI-R scales and sub-scales, showing differential levels of death literacy across dimensions for both the unweighted and weighted sample.

**Table 2.** DLI-R scale and sub-scale scores.

Sample	Unweighted		Weighted	
	DLI-R score	SD	DLI-R score	SD
DLI-R Overall Score	6.06	1.87	6.01	1.85
**DLI-R Scale and Sub-Scale Scores**				
Practical knowledge Scale	6.11	2.00	6.05	1.98
Talking Support Sub-Scale	6.39	2.25	6.34	2.26
Providing Hands on Care Sub-Scale	5.83	2.45	5.77	2.44
Experiential Knowledge Scale	7.20	2.00	7.15	1.98
Factual Knowledge Scale	5.41	2.60	5.36	2.60
Community knowledge Scale	5.52	2.36	5.49	2.64
Accessing Help Sub-Scale	5.19	2.66	5.16	2.64
Support Groups Sub-Scale	5.84	2.39	5.81	2.35

### Multiple linear regression analysis

Table 3 presents the results of the unadjusted, minimally adjusted, and fully adjusted regression models examining associations between sociodemographic and experiential exposure variables, and DLI-R total scores using the weighted sample.

**Table 3.** Linear regression models estimating the association between sociodemographic and experiential exposure factors and death literacy index-revised scores.

Dependent variable: DLI-R score						
Model	Unadjusted (n=2106)		Minimally adjusted (age & gender) (n=2106)		Fully adjusted (n =2106)	
	B	95% CI	B	95% CI	B	95% CI
**Age (ref = 65+)**						
18-24	-0.20	-0.46, 0.06	-0.21	-0.47, 0.05	-0.00	-0.30, 0.30
25-34	0.63	0.39, 0.87***	0.62	0.38, 0.87***	0.45	0.18, 0.71***
35-44	0.02	-0.25, 0.28	0.01	-0.25, 0.28	-0.02	-0.29, 0.24
45-54	0.05	-0.20, 0.30	0.05	-0.20, 0.30	0.14	-0.11, 0.39
55-64	-0.01	-0.24, 0.23	-0.01	-0.25, 0.23	0.01	-0.22, 0.25
**Gender (ref = Female)**						
Male	-0.06	-0.21, 0.10	-0.07	-0.22, 0.08	-0.08	-0.22, 0.07
**Ethnicity (ref = White)**						
Mixed/Multiple Background	-0.43	-0.97, 0.12	-0.50	-1.06, 0.07	-0.48	-1.03, 0.06
Asian or Asian British	0.03	-0.28, 0.34	0.07	-0.26, 0.40	-0.16	-0.46, 0.15
Black or Black British	1.29	0.95, 1.63***	1.06	0.69, 1.42***	0.71	0.35, 1.08**
Other Ethnic Group	-0.47	-0.95, 0.01	-0.54	-1.01, -0.06	-0.55	-1.09, -0.02
**Marital Status (ref = Married)**						
Single and never Married	-0.69	-0.89, -0.49***	-0.79	-1.00, -0.58***	-0.61	-0.82, -0.41***
In a relationship, but not living together	-0.89	-1.24, -0.54***	-1.03	-1.39, -0.67***	-0.88	-1.24, -0.52***
Living with a partner, but not married	-0.45	-0.68, -0.22***	-0.54	-0.78, -0.30***	-0.39	-0.63, -0.15**
Divorced or separated	-0.46	-0.73, -0.19***	-0.34	-0.61, -0.07*	-0.32	-0.59, -0.05*
Widowed	-0.05	-0.44, 0.35	0.11	-0.30, 0.52	-0.01	-0.41, 0.38
Prefer not to say	-1.25	-2.37, -0.13*	-1.37	-2.51, -0.24*	-1.22	-2.14, -0.30**
**Educational Attainment (ref = Below Degree Level)**						
Above Degree Level	0.42	0.26, 0.58***	0.34	0.17, 0.50***	0.14	-0.02, 0.30
**Exposure to End-of-Life Experiences (ref = No Exposure)**						
Personal/Family Exposure	0.73	0.56, 0.89***	0.74	0.58, 0.90***	0.69	0.53, 0.85***
Professional/Volunteer Exposure	1.51	1.27, 1.75***	1.49	1.24, 1.73***	1.38	1.14, 1.63***

*Note.* ***p<0.001; **p<0.01; *p<0.05.

Compared to the reference group (65 years and over), people aged 25–34 had statistically significantly higher DLI-R scores in the unadjusted model, and this association was attenuated but remained statistically significant in the minimally and fully adjusted models. Gender was not significantly associated with death literacy in any of the models.

Compared to the ‘White or White British’ reference group, the ‘Black or Black British’ group had statistically significant higher DLI-R scores, which attenuated but remained statistically significant in the minimally and fully adjusted models.

Compared to those who were married, most non-married groups and those in the ‘prefer not to say’ group had lower death literacy scores in the unadjusted model. For all groups apart from the ‘divorced/separated’ group, the effects were strengthened after adjusting for age and sex in the minimally adjusted model and then attenuated in the fully adjusted model. Compared with the married group, DLI-R scores were not significantly different for those who were widowed.

Educational attainment was positively associated with death literacy in the unadjusted model and after adjusting for age and gender, but this association was no longer statistically significant in the fully adjusted model.

Experiential exposure to end-of-life experiences showed positive associations with death literacy. Compared with having no exposure, ‘personal/family exposure’ and ‘professional/volunteer exposure’ were significantly associated with higher death literacy scores in the unadjusted model. These associations were attenuated after adjustment for age and gender and further attenuated in the fully adjusted model but remained statistically significant.

### Sensitivity analysis

Descriptive characteristics for the 35 respondents who scored the maximum DLI-R score are presented in Supplementary Appendix 3. People aged 25–34, accounted for over one third of maximum scorers.

In our sensitivity analysis where we reran our models excluding these 35 respondents (Supplementary Appendix 4), we found that participants aged 25–34 continued to have statistically significantly higher DLI-R scores than those aged 65 and over, but compared to our main analysis, the size of this association was reduced. This attenuation suggests that extreme scores among younger adults contributed to, but did not fully account for, the higher death literacy observed in this age group.

Differences were also observed for educational attainment and marital status. Educational attainment of ‘degree level and above’ remained statistically significant in the fully adjusted sensitivity model, in contrast to the main analysis. For marital status, the lower DLI-R score for respondents who were divorced or separated attenuated and was no longer statistically significant in the fully adjusted model.

## Discussion

This study provides the first large-scale, nationally representative assessment of death literacy in the UK using the DLI-R. Experiential exposure to end-of-life experiences was associated with higher death literacy, with personal, family, and professional or volunteer experiences all linked to higher DLI-R scores. Death literacy was also associated with age, marital status, and ethnicity in the fully adjusted models, while gender and educational attainment were not. These findings extend previous UK studies^9^ by providing a population-level DLI-R score and by further examining the associations with death literacy across a large nationally representative sample.

The mean DLI-R score in this study (6.01) is higher than those reported in earlier international studies^13,14^ and substantially higher than the 2020 UK-based benchmark score of 4.76.^9^ One potential reason for this difference is that previous studies used the original DLI, whereas we used the DLI-R. The DLI-R is a refined version of the DLI and has demonstrated similar psychometric properties as the original instrument.^14^ Therefore, it may be more likely that differences in sampling and timing contributed to the higher average score in our study. It is also possible that online panels may attract individuals more engaged with health and social issues, potentially including topics related to death and dying, which could contribute to higher reported death literacy. Broader contextual factors may also be relevant. Most death literacy research has occurred during or after the COVID-19 pandemic, a period of heightened public exposure to death and bereavement.^9,13,14^ While such exposure alone is unlikely to increase death literacy, experiential learning theory suggests that capability develops through cycles of experience, reflection and application,^23^ and recent public health education research demonstrates that experiential learning can effectively build health-promotion competencies.^24^ It is therefore plausible that widespread exposure during the pandemic, combined with opportunities for social and personal processing, contribute to higher DLI-R scores in our study compared to in 2020. Despite differences in overall scores, the patterns of scale and sub-scale scores mirrors earlier UK findings, with higher scores for Experiential Knowledge and lower scores for Accessing Help.^9^ This suggests that while overall levels of death literacy may shift over time, relative strengths and gaps across domains may be more stable.

Experiential exposure emerged as the most consistent association, reinforcing the conceptualisation of death literacy as a primarily experience-based competency. Respondents with personal/family experience of serious illness or caregiving, and those with professional/volunteer end-of-life roles, had higher death literacy, in line with prior research.^9,25^ This indicates that such encounters provide practical knowledge, relational skills, and confidence in navigating systems of care.^1,4^ The present findings provide further empirical support for the theorised relationship between direct experiences of end-of-life situations and higher death literacy. From a public health perspective, these findings highlight the importance of experiential learning opportunities, such as caregiving, volunteering, and community engagement, in cultivating death literacy and strengthening compassionate communities.^26,27^ The association between experiential exposure and death literacy aligns with evidence that community participation and caregiving roles build individual and collective competence in engaging with death, dying, and bereavement.^4,8^ This also suggests that death literacy may develop not only through direct involvement but through learning from others’ experiences, including via social networks, community storytelling, and digital media as indicated in prior public health education literature.^28,29^

Marital status was also associated with DLI-R scores, with most non-married groups reporting lower death literacy than married respondents. Social connectedness and relational networks have been identified as important facilitators of practical competence and emotional confidence in end-of-life and bereavement contexts.^30,31^ It is therefore plausible that individuals with fewer or less established relational ties may have fewer opportunities to engage in shared end-of-life care or bereavement experiences. Prior research has found that widowed individuals tend to report higher levels of death literacy.^8,25^ However, in the present study, widowed participants had DLI-R scores comparable to those who were married, and widowhood was not independently associated with death literacy. This finding suggests that both sustained partnership and experiences of loss may contribute to death literacy development through different but overlapping relational and experiential pathways, consistent with conceptualisations of death literacy as a socially situated capability. Associations between death literacy and marital status persisted after adjusting for experiential exposure, indicating that relational position may influence death literacy beyond the end-of-life experiences captured in this study. For example, sustained partnership may provide ongoing opportunities for communication, shared decision-making, and informal caregiving,^32^ which may in turn strengthen confidence and familiarity with death, dying, and bereavement.

Unlike other international studies,^8,10,33^ but consistent with earlier UK findings, ^9^ younger adults (25–34) had higher death literacy than the oldest age group. Taken together, these findings suggest that age may relate to death literacy differently in the UK compared with other countries. This may reflect changing household structures, including increased multigenerational living driven by housing and economic pressures,^34^ which may bring younger adults into closer proximity with illness, caregiving, and bereavement. Notably, in parts of East and Southeast Asia, intergenerational co-residence remains a common household arrangement,^35^ potentially embedding exposure to ageing, illness, and caregiving across the life course. This offers a useful comparative context for understanding how increasing multigenerational living in the UK may shape patterns of exposure to end-of-life experiences. However, population-level studies using the DLI in these settings are currently scarce, and future comparative research would be valuable in examining whether similar age-related patterns in death literacy are observed.

Ethnic differences in death literacy were observed across models, with the Black or Black British group demonstrating higher DLI-R scores compared with the White reference group. Prior research indicates that some minority ethnic communities in the UK are more likely to provide family-based end-of-life care and less likely to be able to access formal hospice or care home services.^36,37^ In addition, people from minority ethnic backgrounds are more likely to be represented within parts of the health and social care workforce in the UK, which may provide further opportunities for exposure to end-of-life care through professional roles.^38^ Cultural practices and norms around family responsibility, collective caregiving, and mourning may also shape how knowledge and confidence related to death and dying are developed and shared.^39^ Together, these findings suggest that the observed association between ethnicity and death literacy in this sample may reflect a complex interplay of cultural, structural, occupational, and experiential factors, rather than ethnicity acting as an independent association.

Finally, educational attainment and gender were not associated with death literacy in the fully adjusted main model. Although higher educational attainment was positively associated with DLI-R scores in unadjusted analyses, this relationship attenuated after adjustment for all other variables. These findings are consistent with previous studies, which similarly reported no clear differences in death literacy by gender or educational attainment.^8,9,25^ Importantly, this pattern contrasts with research on health literacy, where formal educational attainment and gender are often strongly associated.^40,41^ The absence of comparable associations here reinforces the conceptualisation of death literacy as a capability shaped primarily through experience, social participation, and exposure to end-of-life contexts, rather than through formal education or demographic positioning alone.

### Strengths and limitations

A key strength of this study is its large, nationally representative sample, enabling generalisable estimates of death literacy in the UK. The use of the DLI-R supports cross-study comparability.^14^ Nevertheless, several limitations should be acknowledged. The cross-sectional design precludes causal inference, meaning that observed associations between sociodemographic and experiential variables and death literacy can only be interpreted as correlational. In addition, the study utilised the DLI-R, which is a self-reported measure, which may introduce bias. The use of an online panel provider enabled rapid collection of data from a representative population. Because the aim of using the panel provider was sample representativeness, a power calculation was not conducted a priori, meaning the study may have been underpowered to detect smaller effect sizes, though the sample size was sufficient for the multivariable regression analysis conducted. Despite weighting, online data collection may under-represent populations with limited digital access, and participation in panel-based surveys may attract respondents who are more engaged with or comfortable reflecting on issues related to death and dying, potentially influencing observed levels of death literacy. Smaller subgroup sizes also mean that results pertaining to some groups should be interpreted with caution. Moreover, whilst a broad range of variables were included, other unmeasured factors may play a role in shaping death literacy that were not examined in this study. Finally, though this study focused on total DLI-R scores rather than examining its scales independently, previous research suggests that the associations of death literacy may differ across domains, for example, factors influencing experiential knowledge may not mirror those that shape factual knowledge.^10,14^ Future analyses disaggregating subscale-level effects would provide a more nuanced understanding of how death literacy is distributed and developed within the population.

### Implications for practice and research

The findings of this study underscore the importance of experiential exposure, through both personal/family and professional/volunteer contexts, as a key association with death literacy. Interventions seeking to enhance public competence and confidence around death and dying might therefore prioritise opportunities for experiential engagement, peer learning, and community-based dialogue. This aligns with Compassionate Communities and public health palliative care frameworks that emphasise participation, relational learning, and local capacity-building.^26^ From a research perspective, further studies are warranted to explore domain-specific predictors of death literacy and to examine how these evolve over time. Longitudinal and interventional designs would enable a clearer understanding of temporality and causal direction, as well as the effects of targeted educational, community, and policy initiatives. Building on previous calls for comparative and cross-contextual analyses,^25^ future research should also test the generalisability of these UK findings across cultural settings and evaluate whether different social and policy environments shape how death literacy develops and is enacted. An important additional priority is to move beyond binary measures of exposure to examine how the quality, context, and emotional impact of end-of-life experiences influence the development of death literacy, as exposure alone may not be uniformly beneficial. Further research is also needed to explore how death literacy is conceptualised, expressed, and supported across diverse ethnic and community contexts within the UK, including how structural inequalities and differential access to services shape opportunities for learning through experience. Collectively, such work could strengthen the evidence base underpinning death literacy as both a measurable construct and a practical lever for improving end-of-life preparedness, care, and community capacity to engage with issues of dying, death, and bereavement.

## Conclusion

This nationally representative study extends prior UK research by providing the most comprehensive population-level measure of death literacy to date and identifying characteristics and experiences associated with higher or lower levels. Experiential exposure to end-of-life contexts, whether personal, familial, professional or voluntary, emerged as the strongest correlate of higher death literacy, alongside differences by marital status, ethnicity, and age. These results highlight the importance of lived experience, social participation, and community connection in shaping public understanding and engagement with death, dying, and bereavement. Strengthening such experiential, relational, and support-pathway opportunities within communities may be central to enhancing the UK’s collective capacity to live and die well.

## Supplemental material

Supplemental material (Appendices 1-3) - Associations between sociodemographic factors, experience of death and dying, and death literacy: A nationally representative cross-sectional study Supplemental material (Appendices 1-3) for Associations between sociodemographic factors, experience of death and dying, and death literacy: A nationally representative cross-sectional study by Melanie F. J. Diggle, Therese Johansson, Joanna M. Davies, Kate Heaps, Katherine E. Sleeman in Palliative Care and Social Practice

## Data Availability

The datasets generated and/or analysed during the study are available from the corresponding author upon reasonable request.*
