# Supplementary material for: Associations between sociodemographic factors, experience of death and dying, and death literacy: A nationally representative cross-sectional study
Source: Palliat Care Soc Pract. 2026 Sep 3;20:26323524261473937. doi: 10.1177/26323524261473937 (PMC13546418; doi:10.1177/26323524261473937)
Supplement: Supplementary file 1 [file sj-pdf-1-pcr-10.1177_26323524261473937.pdf]

Supplementary Material

Appendix 1:

Checklist for Reporting Results of Internet E-Surveys (CHERRIES)

Checklist Item	*Explanation*	*Page Number*
Describe survey design	Describe target population, sample frame. Is the sample a convenience sample? (In “open” surveys this is most likely.)	4
IRB approval	Mention whether the study has been approved by an IRB.	7
Informed consent	Describe the informed consent process. Where were the participants told the length of time of the survey, which data were stored and where and for how long, who the investigator was, and the purpose of the study?	5
Data protection	If any personal information was collected or stored, describe what mechanisms were used to protect unauthorized access.	5
Development and testing	State how the survey was developed, including whether the usability and technical functionality of the electronic questionnaire had been tested before fielding the questionnaire.	4-5
Open survey versus closed survey	An “open survey” is a survey open for each visitor of a site, while a closed survey is only open to a sample which the investigator knows (password-protected survey).	5
Contact mode	Indicate whether or not the initial contact with the potential participants was made on the Internet. (Investigators may also send out questionnaires by mail and allow for Web-based data entry.)	5
Advertising the survey	How/where was the survey announced or advertised? Some examples are offline media (newspapers), or online (mailing lists – If yes, which ones?) or banner ads (Where were these banner ads posted and what did they look like?). It is important to know the wording of the announcement as it will heavily influence who chooses to participate. Ideally the survey announcement should be published as an appendix.	4-5
Web/E-mail	State the type of e-survey (e.g., one posted on a Web site, or one sent out through e-mail). If it is an e-mail survey, were the responses entered manually into a database, or was there an automatic method for capturing responses?	5
Context	Describe the Web site (for mailing list/newsgroup) in which the survey was posted. What is the Web site about, who is visiting it, what are visitors normally looking for? Discuss to what degree the content of the Web site could pre-select the sample or influence the results. For example, a survey about vaccination on a anti-	N/A

	immunization Web site will have different results from a Web survey conducted on a government Web site	
Mandatory/voluntary	Was it a mandatory survey to be filled in by every visitor who wanted to enter the Web site, or was it a voluntary survey?	5
Incentives	Were any incentives offered (e.g., monetary, prizes, or non-monetary incentives such as an offer to provide the survey results)?	5
Time/Date	In what timeframe were the data collected?	5
Randomization of items or questionnaires	To prevent biases items can be randomized or alternated.	5
Adaptive questioning	Use adaptive questioning (certain items, or only conditionally displayed based on responses to other items) to reduce number and complexity of the questions.	5
Number of Items	What was the number of questionnaire items per page? The number of items is an important factor for the completion rate.	4
Number of screens (pages)	Over how many pages was the questionnaire distributed? The number of items is an important factor for the completion rate.	4
Completeness check	It is technically possible to do consistency or completeness checks before the questionnaire is submitted. Was this done, and if "yes", how (usually JAVAScript)? An alternative is to check for completeness after the questionnaire has been submitted (and highlight mandatory items). If this has been done, it should be reported. All items should provide a non-response option such as "not applicable" or "rather not say", and selection of one response option should be enforced.	4-5
Review step	State whether respondents were able to review and change their answers (e.g., through a Back button or a Review step which displays a summary of the responses and asks the respondents if they are correct).	5
Unique site visitor	If you provide view rates or participation rates, you need to define how you determined a unique visitor. There are different techniques available, based on IP addresses or cookies or both.	5
View rate (Ratio of unique survey visitors/unique site visitors)	Requires counting unique visitors to the first page of the survey, divided by the number of unique site visitors (not page views!). It is not unusual to have view rates of less than 0.1 % if the survey is voluntary.	Data not available from the individual panel providers
Participation rate (Ratio of	Count the unique number of people who filled in the first survey page (or agreed to participate, for example by checking a checkbox),	Unable to calculate

unique visitors who agreed to participate/unique first survey page visitors)	divided by visitors who visit the first page of the survey (or the informed consents page, if present). This can also be called “recruitment” rate.	
Completion rate (Ratio of users who finished the survey/users who agreed to participate)	The number of people submitting the last questionnaire page, divided by the number of people who agreed to participate (or submitted the first survey page). This is only relevant if there is a separate “informed consent” page or if the survey goes over several pages. This is a measure for attrition. Note that “completion” can involve leaving questionnaire items blank. This is not a measure for how completely questionnaires were filled in. (If you need a measure for this, use the word “completeness rate”.)	7
Cookies used	Indicate whether cookies were used to assign a unique user identifier to each client computer. If so, mention the page on which the cookie was set and read, and how long the cookie was valid. Were duplicate entries avoided by preventing users access to the survey twice; or were duplicate database entries having the same user ID eliminated before analysis? In the latter case, which entries were kept for analysis (e.g., the first entry or the most recent)?	5
IP check	Indicate whether the IP address of the client computer was used to identify potential duplicate entries from the same user. If so, mention the period of time for which no two entries from the same IP address were allowed (e.g., 24 hours). Were duplicate entries avoided by preventing users with the same IP address access to the survey twice; or were duplicate database entries having the same IP address within a given period of time eliminated before analysis? If the latter, which entries were kept for analysis (e.g., the first entry or the most recent)?	5
Log file analysis	Indicate whether other techniques to analyze the log file for identification of multiple entries were used. If so, please describe.	5
Registration	In “closed” (non-open) surveys, users need to login first and it is easier to prevent duplicate entries from the same user. Describe how this was done. For example, was the survey never displayed a second time once the user had filled it in, or was the username stored together with the survey results and later eliminated? If the latter, which entries were kept for analysis (e.g., the first entry or the most recent)?	5
Handling of incomplete questionnaires	Were only completed questionnaires analyzed? Were questionnaires which terminated early (where, for example, users did not go through all questionnaire pages) also analyzed?	7
Questionnaires submitted	Some investigators may measure the time people needed to fill in a questionnaire and exclude questionnaires that were submitted too	5

with an atypical timestamp	soon. Specify the timeframe that was used as a cut-off point and describe how this point was determined.	
Statistical correction	Indicate whether any methods such as weighting of items or propensity scores have been used to adjust for the non-representative sample; if so, please describe the methods.	4

This checklist has been modified from Eysenbach G. Improving the quality of Web surveys: the Checklist for Reporting Results of Internet E-Surveys (CHERRIES). J Med Internet Res. 2004 Sep 29;6(3):e34 [erratum in J Med Internet Res. 2012; 14(1): e8.]. Article available at https://www.jmir.org/2004/3/e34/; erratum available https://www.jmir.org/2012/1/e8/. Copyright ©Gunther Eysenbach. Originally published in the Journal of Medical Internet Research, 29.9.2004 and 04.01.2012.

This is an open-access article distributed under the terms of the Creative Commons Attribution License (https://creativecommons.org/licenses/by/2.0/), which permits unrestricted use, distribution, and reproduction in any medium, provided the original work, first published in the Journal of Medical Internet Research, is properly cited.

Appendix 2:

Death Literacy Index – Revised[12]

1. Practical knowledge (2 Sub-Scales)

1.1 Talking Support

Conversations about dying, death, or grief.

How able are you to have the following conversations, where 1= not at all able to 5= very able? Please tick.

1. Talking about dying, death, or grief with a close friend
2. Talking about dying, death, or grief to a child
3. Talking to a grieving person about their loss
4. Talking to a healthcare professional about getting support for a dying person where they live.

1.2 Providing Hands on Care

How able are you to do the following, where 1= not at all able to 5 = very able? Please tick.

5. Feed or help a person to eat
6. Wash a person
7. Lift a person or help them move
8. Administer injections

2. Experiential Knowledge

To what extent do you agree with the following? Indicate your response on a scale of 1-5, where 1=do not agree at all and 5=strongly agree.

Previous experience of grief, losing someone, or other important life events have…

9. Made me more emotionally prepared to support others with death, dying and bereavement
10. Made me think about what is important and not important in life
11. Developed my wisdom and understanding
12. Made me more compassionate toward myself
13. Made me better prepared to face similar challenges in the future

3.Factual Knowledge

To what extent do you agree with the following? Indicate your response on a scale of 1-5, where 1=do not agree at all and 5=strongly agree.

14. I know the rules and regulations when a person dies at home
15. I know what documents are needed when planning for death
16. I know enough about the healthcare system to find the support that a dying person needs
17. I know enough to make decisions about funeral services and options
18. I know how to access palliative care in my area
19. I know enough about how illnesses progress to make decisions about medical treatments at the end of life
20. I know about different ways that funeral home staff can be of help around funerals (adapted for UK audience based on work by Graham-Wisener et al., 2022)

4. Community Knowledge (2 Sub-Scales)

4.1 Others can help me provide end of life care.

Please rate your level of agreement with the following statements. If I were to provide end of life care for someone, I know people who could help me (on a scale of 1-5 between Strongly disagree to Strongly agree)

21. To get support in the area where I live e.g., from clubs, associations, or volunteer organisations
22. To get help with providing day-to-day care for a person at the end of life
23. To get equipment that is required for care
24. To get support that is culturally appropriate for a person
25. To get emotional support for myself

4.2 Support groups in my community

Please rate your level of agreement with the following statements. There is support in my community for (on a scale of 1-5 between Strongly disagree to Strongly agree)

26. People with life-threatening illnesses
27. People who are nearing the end of their lives
28. People who caring for a dying person
29. People who are grieving

Appendix 3:

Sample characteristics for maximum DLI-R scorers

	n	%
**Age Group**		
18-24	1	3.9
25-34	13	38.1
35-44	8	22.3
45-54	8	22.2
55-64	4	11.0
65+	1	2.6
**Gender**		
Male	17	48.7
Female	18	51.3
**Ethnicity**		
White or White British	27	80.2
Mixed/Multiple Background	0	0
Black or Black British	3	10.2
Asian or Asian British	3	9.6
Other	0	0
**Educational Attainment**		
Below degree qualifications	21	61.2
Degree level or above qualifications	13	38.8
**Marital Status**		
Single and never married	7	19.1
In a relationship, but not living together	0	0
Living with a partner but not married	4	11.1
Married	23	66.8
Divorced or separated	0	0
Widowed	1	3.0
Prefer not to say	0	0
**Exposure to End-of-Life Experiences**		
Personal/Family Exposure	8	22.9
Professional/Volunteer Exposure	8	24.4
None of these	18	52.7

Appendix 4:

Sensitivity Analysis: Linear regression models estimating the association between sociodemographic and experiential factors and Death Literacy Index-Revised scores (Excluding Top Scorers)

Dependent Variable: DLI-R Score						
Model	Unadjusted (n=2,071)		Minimally Adjusted (Age & Gender) (n=2,071)		Fully Adjusted (n=2,071)	
	B	95% CI	B	95% CI	B	95% CI
Age (ref = 65+)						
18-24	-0.21	-0.47, 0.04	-0.22	-0.48, 0.03	-0.04	-0.34, 0.26
25-34	0.50	0.27, 0.74***	0.50	0.26, 0.74***	0.33	0.07, 0.60*
35-44	-0.07	-0.33, 0.19	-0.07	-0.33, 0.19	-0.11	-0.37, 0.15
45-54	-0.03	-0.27, 0.22	-0.03	-0.27, 0.22	0.05	-0.20, 0.29
55-64	-0.04	-0.27, 0.19	-0.05	-0.28, 0.19	-0.03	-0.25, 0.21
Gender (ref = Female)						
Male	-0.06	-0.21, 0.09	-0.08	-0.23, 0.08	-0.08	-0.22, 0.07
Ethnicity (ref = White)						
Mixed/Multiple Background	-0.37	-0.91, 0.18	-0.42	-0.98, 0.14	-0.42	-0.96, 0.13
Asian or Asian British	0.00	-0.30, 0.30	0.05	-0.26, 0.37	-0.15	-0.45, 0.16
Black or Black British	1.19	0.86, 1.52***	1.00	0.64, 1.35***	0.65	0.30, 0.99***
Other Ethnic Group	-0.41	-0.87, 0.07	-0.46	-0.93, 0.02	-0.47	-0.99, 0.06
Marital Status (ref = Married)						
Single and never Married	-0.65	-0.84, -0.46***	-0.74	-0.95, -0.54***	-0.57	-0.77, -0.37***
In a relationship, but not living together	-0.80	-1.15, -0.46***	-0.93	-1.29, -0.57***	-0.79	-1.15, -0.43***
Living with a partner, but not married	-0.42	-0.64, -0.19***	-0.50	-0.73, -0.27***	-0.35	-0.58, -0.12**
Divorced or separated	-0.37	-0.63. -0.10**	-0.27	-0.55, -0.00*	-0.26	-0.52, 0.01
Widowed	0.00	-0.39, 0.39	0.12	-0.29, 0.52	-0.00	-0.39, 0.39
Prefer not to say	-1.16	-2.28, -0.04*	-1.29	-2.42, -0.17*	-1.18	-2.08, -0.27*
Educational Attainment (ref = Below Degree Level)						
Above Degree Level	0.41	0.25, 0.57***	0.35	0.19, 0.52***	0.17	0.01, 0.33*
Exposure to End-of-Life Experiences (ref = No Exposure)						
Personal/Family Exposure	0.74	0.58, 0.90***	0.75	0.59, 0.90***	0.70	0.54, 0.85***
Professional/Volunteer Exposure	1.46	1.22, 1.69***	1.46	1.21, 1.70***	1.35	1.11, 1.60***

Note: ***p<0.001; **p<0.01; *p<0.05
